# Russell Body Gastritis: an Unusually Presentation of the Chronic Gastritis

**Published:** 2017-01-19

**Authors:** Betül CENGIZ PEKER, Fatma Secil KIRDOK, Hayrettin DIZEN

**Affiliations:** 1 *Department of Pathology Yunus Emre State Hospital, Eskisehir, Turkey*; 2 *Department of Gastroenterology, Yunus Emre State Hospital, Eskisehir, Turkey*; 3 *Department of Surgery, Yunus Emre State Hospital, Eskisehir, Turkey*

**Keywords:** Russell Body, Gastritis, *Helicobacter pylori*

## Abstract

Russell body gastritis is a rare form of chronic gastritis. It is characterized by the invasion of lamina propria by plasma cells that included eosinophilic cytoplasmic inclusion. In the literature, most of the cases are associated with Helicobacter pylori. Russell body gastritis and Helicobacter pylori infection are generally seen together incidentally. We report here two cases of Russell body gastritis with *Helicobacter* *pylori* infection in a 51-yr-old woman and a 39-yr-old man from *Eskisehir, Turkey.*

## Introduction

Plasma cells are mixed with lymphocytes and a variable amount of neutrophils through gastric lamina propria in chronic gastritis. Russell body gastritis was defined to localize the accumulation of plasma cells containing Russell bodies in gastric mucosa. These plasma cells with Russell bodies are defined as Mott cells. They are generally identified in the setting of different malignancies of hematopoietic origin. However, it is possible to see them in some reactive conditions. Diagnostic pathologists should be careful while the diagnosis. Russell body gastritis is a rare form of chronic gastritis (1). However, the etiology of Russell body gastritis is still unclear today.

We report here two cases of Russell body gastritis associated with *Helicobacter pylori* infection confirmed by clinical, morphological and immunohistochemical studies in a 51-yr-old woman and a 39-yr-old man from *Eskisehir, Turkey*

## Case report

**Case 1**

**Fig. 1:**
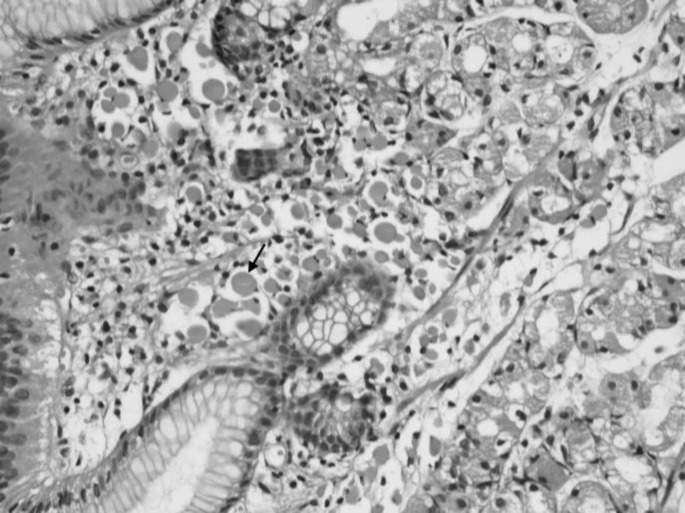
Characteristic appearance of Russell body gastritis. The lamina propria is expanded by Mott cells (H§E; X400

**Fig. 2:**
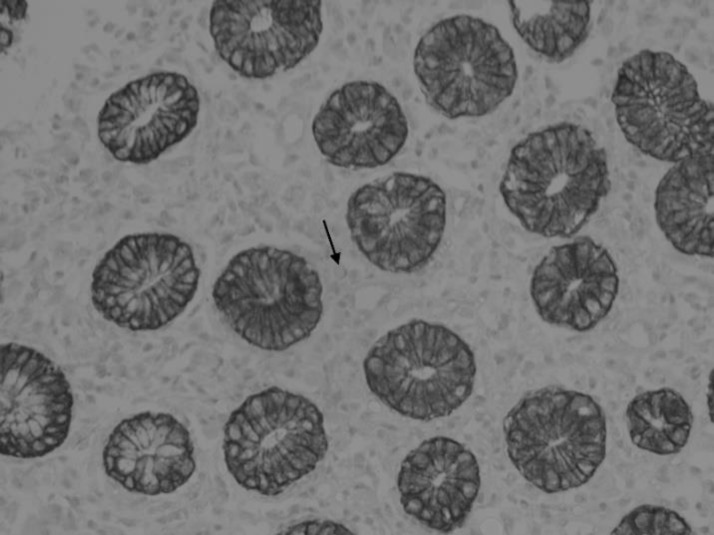
The Mott cells are negative for cytokeratin (X400

A 51-year-old woman presented with dyspeptic symptoms. Physical examination and laboratory data did not reveal any abnormalities. Diffuse hyperemic and edema were detected by upper gastrointestinal endoscopy in all gastric mucosa. Esophageal was normal. The biopsies were taken from the corpus region. Histological examination was observed Mott cells in the lamina propria with chronic gastritis findings (Fig. 1). Mott cells stained positive for CD138 and negative for cytokeratin (Fig. 2). Kappa and Lambda light chains showed a polyclonal origin of plasma cells.

Giemsa staining for *H. pylori* infection in the corpus biopsies materials was positive. After eradication of the *H. pylori* with antibiotics, endoscopic controls were recommended to the patient. Following-up upper gastrointestinal endoscopy and biopsy will be planned. Informed consent was taken from the patient.

**Case 2**

**Fig 3:**
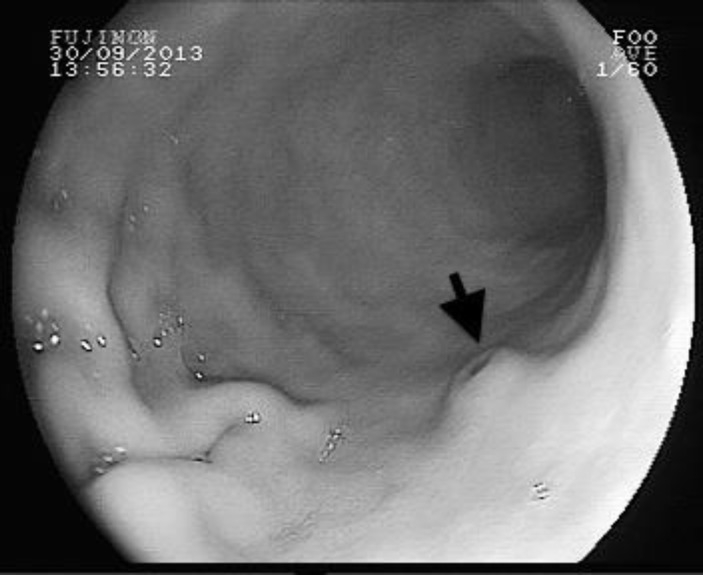
Endoscopic view of patient 2, polypoid formation with small ulceration was detected on the distal of corpus

**Fig.4:**
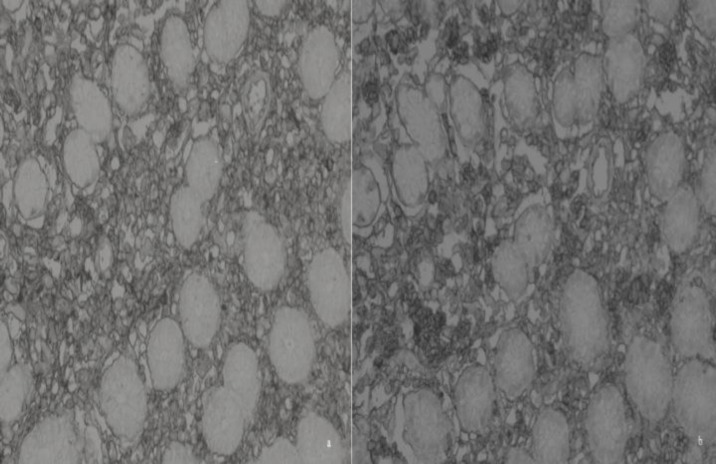
Kappa (a) and Lambda (b) chains are diffusely immunoreactive in the Mott cells (X400

A 39-year-old man presented with epigastric pain. Physical examination and laboratory data did not show any abnormalities. Upper gastrointestinal endoscopy revealed hyperemic antral mucosa. In addition, polypoid formation with small ulceration was detected on the distal of corpus (Fig. 3). The biopsies were taken from the corpus region. Histological examination of the biopsy revealed gastric mucosa with diffuse plasma cell infiltration in the lamina propria, associated with Russell bodies. Mott cells stained positive for CD138 and negative for cytokeratin. Kappa and Lambda light chains showed a polyclonal pattern of plasma cells (Fig. 4). Informed consent was taken from the patient.

Giemsa staining for *H. pylori* infection in the corpus biopsies materials was positive.

*H. pylori* eradication treatment was administered to the patient. After that, post-eradication endoscopic controls and follow-up upper gastrointestinal endoscopy and biopsy were recommended to the patient.

## Discussion

Russell bodies were first described in 1890 (2). These Russell bodies arise because of abnormal functioning of the normal immunoglobulin secretion pathways in plasma cells (3). These plasma cells with Russell bodies can be seen in the chronic inflammation of the gastrointestinal mucosa. They are generally identified in the setting of different diseases such as in multiple myeloma, malignant lymphoma, Hashimato’s thyroiditis, rheumatoid arthritis, ulcerative colitis, and some malignant cells of plasmocytoma (3, 4).

In 1998, Tazawa and Tsutsumi (1) have first used Russell body gastritis. They reported the accumulation of plasma cells containing Russell bodies as secondary to *H. pylori* infection (1). The reason for the localized accumulation plasma cells is unclear. On the other hand, chronic antigenic stimulation caused by *H. pylori* infection is suspected as a reason. But, in the literature HIV positive Russell body gastritis and *H. pylori* negative Russell body gastritis was encountered (5, 6). 

The individuals affected by Russell body gastritis are generally women (5). Most of the patients referred with non-specific gastrointestinal complaints. These symptoms are abdominal discomfort, nausea, and dyspepsia. Endoscopic features, such as symptoms, are nonspecific such as in mucosal erythema, edema, erosion, and ulceration. Sometimes, raised nodules can be seen (3). Russell body gastritis is thought to be a focal condition. Therefore, multiple endoscopic biopsies should be performed.

Russell body gastritis is confirmed with microscopic findings. The diagnosis of Russell body gastritis is made when numerous Mott cells appear in a gastric mucosa. 

The differential diagnosis of Russell body gastritis includes plasmocytoma, MALToma and signet ring carcinoma. Cytokeratin negativity rules out the signet-ring carcinoma. The polyclonal structure of plasma cell infiltration is represented by the kappa and lambda chain expression. The kappa and Lambda polyclonal immunoreactive pattern exclude MALToma and plasmocytoma (7). In recent years, monoclonal Mott cell proliferation has been reported. The monoclonal B cell proliferation associated with chronic inflammation (8). Diagnostic pathologists should be cautious of giving the diagnosis of neoplastic growth (such as MALT lymphoma), just based on the immunohistochemical identification of immunoglobulin light chain restriction. Our two cases showed polyclonality with the kappa and lambda immunohistochemistry stain. Our two cases showed negative immunoreactivity with cytokeratin immunohistochemistry stain. Some Russell body gastiritis show similar features as that of gastiric xanthoma, endoscopically. Histopathologic examination is needed to establish the distinctive diagnosis (2).

In the cases with *Helicobacter*-associated Russell body gastritis, first, *H. pylori* eradication therapy and exclusion of associated conditions should be done. Other associated conditions include other infectious agents (6, 7, 9) ethanol abuse (1, 10) and gastric carcinoma (7, 11). 

## Conclusion

The long-term effects of the localized accumulation of plasma cells are unclear and the etiopathogenesis of this entity is not completely understood. For understanding of pathogenesis and long-term effects in the Russell body gastritis are needed more case reports and studies. 

## Conflict of Interests

The authors declare that there is no Conflict of Interests. 
